# Educative Hybrid Intervention as a Strategy for Reintegration to the Clinical Courses of Undergraduate Students in COVID-19 Pandemic

**DOI:** 10.7759/cureus.15699

**Published:** 2021-06-16

**Authors:** Jorge E Valdez-Garcia, Guillermo Dominguez-Cherit, Eder Luna-Ceron, Alfredo Pherez-Farah, Sebastian Muzquiz-Aguirre, Juan P Mancilla-Ortega, Laura J Vichi-Lima, Sahaira J Montejo-Romo, Rebeca Bonilla-Hernandez, Daniel Arizpe-Vilana, Karina Jimenez-Becerril, Elena Rios-Barrientos, Tania R Garibay-Huarte, Enrique R Jean-Silver, Vianey A Zaragoza-Guerra, Jose A Diaz-Elizondo, Eduardo Rivero-Sigarroa, Lydia Zerón-Gutierrez

**Affiliations:** 1 Department of Clinical Sciences, Tecnológico de Monterrey, Monterrey, MEX; 2 Department of Clinical Sciences, Tecnológico de Monterrey, Mexico City, MEX; 3 Department of Anaesthesiology, Instituto Nacional de Ciencias Médicas y Nutrición "Salvador Zubirán", Mexico City, MEX; 4 Department of Internal Medicine, Centro Médico ABC, Mexico City, MEX; 5 Department of Clinical Sciences, Universidad Popular Autónoma de Puebla, Puebla, MEX; 6 Department of Clinical Sciences, Tecnologico de Monterrey, Mexico City, MEX; 7 Department of Medical Simulation, Tecnologico de Monterrey, Monterrey, MEX; 8 Department of Clinical Sciences, Tecnologico de Monterrey, Guadalajara, MEX; 9 Department of Clinical Sciences, Tecnologico de Monterrey, Monterrey, MEX

**Keywords:** covid-19, hybrid education, clinical clerkships, training, skills and simulation training, distance learning programs

## Abstract

The SARS-CoV-2 pandemic generated the need to modify the current clinical educational model with the challenge of promoting safety and the continuity of clinical education through the use of virtual platforms. Since clinical training in hospital institutions cannot be substituted, a strategic training plan was developed to guarantee protection, safety, and academic continuity for students upon returning to clinical clerkships.

The objective of this project was to develop and evaluate the impact of a massive hybrid training plan as an educative strategy to give the theoretical and practical knowledge required for the safe return of undergraduate students to their respective clinical activities in the context of this pandemic. An academic program was designed through a massive hybrid strategy to train 616 undergraduate students studying clinical cycles by presential, virtual, synchronous, and asynchronous activities.

To know the program's impact, a study based on an initial evaluation and a final evaluation was carried out to evaluate the acquisition of the critical knowledge and skills of the program. A significant difference was found between the means of the initial and final evaluations (p <0.001), as well as a high impact of the intervention (d 1.6). Significant improvements in the areas of COVID-19 initial management (p <0.001) and personal protective equipment use (p <0.001) were seen in the post-test when compared to the initial evaluation. Both a quantitative and a qualitative analysis were carried out, finding positive results on the course design, quality of didactic resources, and instructors' performance. Massive hybrid training is an effective strategy to facilitate the reintegration of undergraduate students into their face-to-face clinical rotations.

## Introduction

The COVID-19 pandemic, declared by the World Health Organization (WHO) in March 2020, has generated an unprecedented impact on the education sector, affecting nearly 1.5 billion students worldwide and more than 37 million in Mexico [1]. Particularly in medical education, the Mexican Association of Faculties and Schools of Medicine (AMFEM) and the Association of American Medical Colleges (AAMC) recommended the temporary suspension of clinical activities given that in addition to compromising the health of undergraduate students, they did not have the necessary skills to face the pandemic [1-4]. The National School of Medicine and Health Sciences (EMCS) of the Tecnológico de Monterrey withdrew the students from hospital and outpatient areas of the degrees of Nutrition and Comprehensive Well-being (LNB), Surgeon Odontologist (MO), and Surgeon (MC) as of March 17, being the first Mexican institution to take this measure [5-8].

Various institutions have implemented strategies to ensure compliance with the objectives of their programs, migrating them from a face-to-face mode to a virtual one. Several observational studies have shown the usefulness of the latter to maintain learning processes. However, student satisfaction rates have been suboptimal [9, 10]. In the United Kingdom, it was evidenced that virtual education limits student participation and that online classes are less effective during the period of clinical rotations, highlighting the importance of contact with patients, which is why the hybrid model has emerged as an alternative [11-15].

According to a study carried out at the University of West Chester, 64% of the students felt more involved with the course content in the hybrid format and 90% found it convenient [15]. Given its effectiveness in promoting greater learning, some of the best universities in the world, such as the Imperial College of London, the Massachusetts Institute of Technology, and the University of Maryland, have incorporated this model into their educational offerings. Under the current context, the main challenge is to define how much learning can be undertaken online and how much clinical exposure is still needed [1, 12, 16]. In this regard, although several institutions around the world began to design hybrid programs for educational continuity, the development of this strategy inside medical education continues to be scarcely studied. 

Given the importance of reintegration into the clinical environment within a “new normal”, universities must design a strategic training plan that guarantees the safety, academic continuity, and emotional support of students, who face educational uncertainty and fear derived from the disease [17-20]. The AAMC published a guide for medical student contact with patients during the pandemic, emphasizing three points: information on COVID-19, proper use and availability of personal protective equipment (PPE), and dynamics of the hospital service, taking into account the systematization of actions and standards necessary to optimize care [7, 21].

At the Tecnológico de Monterrey, after three months of withdrawal of undergraduate students from clinical courses, the possibility of their reincorporation was raised, so a hybrid educational strategy was designed to provide the knowledge and the fundamental skills to achieve an adequate face-to-face reintegration into the clinical environment within the context of COVID-19 pandemic. This program adopted the advantages of the successful hybrid format from other institutions, however, the contents, didactic techniques, and organization were fully developed by our institution alone. The objective of this study was to evaluate the theoretical knowledge, skills, and experiences acquired by health sciences students enrolled in this academic program. 

## Materials and methods

**Figure 1:**
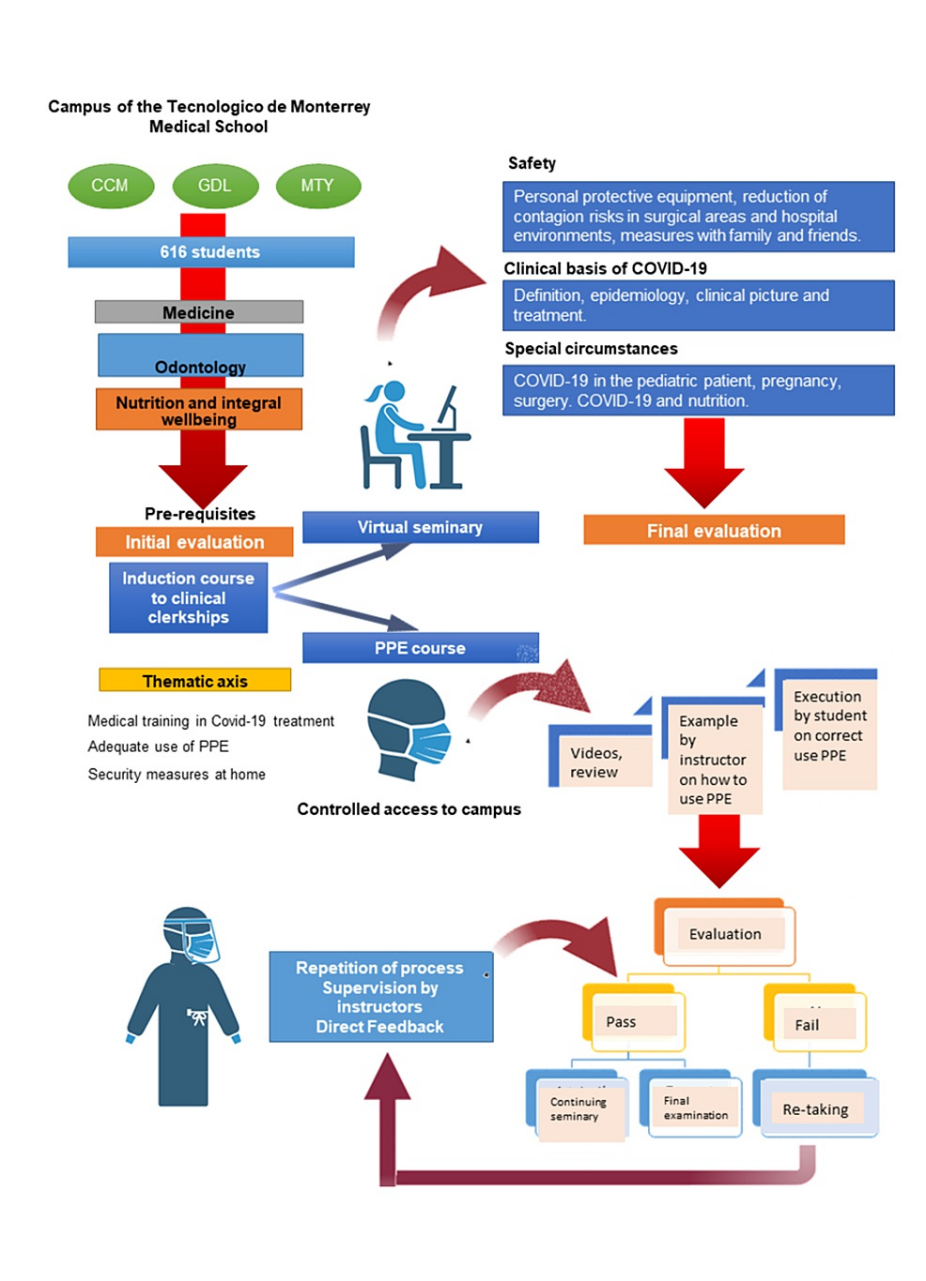
Design of the hybrid educational strategy aimed at undergraduate students in clinical courses. The logistical mechanisms used to carry out the face-to-face workshop on personal protective equipment (PPE) and the thematic axes of the virtual theoretical seminar are distinguished. CCM: Mexico City Campus; GDL: Guadalajara Campus; MTY: Monterrey Campus.

An educational intervention based on a hybrid strategy was designed with three general objectives: medical training in COVID-19, proper use of PPE, and hospital biosecurity measures (Figure 1). The program focused especially on the last two objectives since they are not offered as part of their regular academic curricula. To register, the students had to fulfill a series of prerequisites that consisted of taking general courses on COVID-19 and biosafety. Once passed, they were given access to the course that consisted of a theoretical seminar through the Canvas platform (Instructure Inc., Utah, USA), where the students could access video lectures as well as reading and evaluation materials. Additionally, a face-to-face practical workshop on the use of PPE was given within the medical school facilities. The medical school infrastructure was previously adapted to facilitate the interaction among students and faculty members following set safety measures to prevent viral spread. 

The theoretical seminar was designed in a virtual format and lasted 20 hours, taught over five days by professors from EMCS and the National Institutes of Health, through platforms such as Zoom (Zoom video communication Inc, California, USA) and Microsoft Teams (Microsoft, Washington, USA). Additionally, the workshop on the use of PPE was carried out in simulation spaces and consisted of a theoretical instructional video followed by a practical face-to-face phase of two hours in duration, in which the medical students were able to practice the correct procedure of hand-washing and PPE use.

To explore the impact of this academic program on the acquisition of theoretical knowledge and practical skills, we conducted an initial theoretical evaluation in the first day of instruction, followed by a final theoretical evaluation in the last day of instruction. Additionally, after the completion of the face-to-face workshop for correct use of PPE and hand-washing, a practical examination in a form of a simulation exercise was conducted and evaluated by faculty, and performance was recorded in a summary checklist. Students that did not achieve an adequate proficiency in the correct use of PPE were allowed to re-take the examination. Data for this study was gathered from the initial and final theoretical evaluation forms. Also, data was obtained from performance checklists after the final evaluation of the practical workshop. For the analysis presented in this study, we only considered the results from the first evaluation attempt of each student in any of the examinations. Finally, we performed a satisfaction survey to receive feedback and comments from students. 

A demographic and exploratory analysis was carried out using descriptive statistics. To verify the normality of the distribution of the variables, corresponding to the scores in the initial and final evaluations, the Kolmogorov-Smirnov test was used. To compare the means between these variables, the Wilcoxon W test was performed for non-parametric distribution. To evaluate the size of the impact of this intervention, Cohen's D test was performed. To analyze the change in the proportions of questions answered correctly in the initial and final evaluation, a two-sample z-test of proportions was used. All statistical tests were carried out using StataCorp Stata MP software (Version 14.0, College Station, TX, USA). The results of these analyzes were presented graphically using GraphPad Prism 8 software (Version 8.0, San Diego, CA, USA). The figures were made by using the Biorender platform (Biorender Company, 2017, USA). The qualitative analysis of the feedback from the trained group was done using the MaxQDA software (Version 2020, Berlin, Germany). The ethical protocols required by the research center for the recruitment and evaluation of study subjects were followed, and they were duly informed about the purposes of this study and decided to participate voluntarily.

## Results

During the intervention period, a total of 616 students were trained, of which 60.47% were from Campus Monterrey, 27.85% from Campus Ciudad de México, and 11.68% from Campus Guadalajara. Almost 90% corresponded to the MC career, followed by LNB with 8% and MO with less than 2%. Only 37.17% were in the sixth year, as the duration of the LNB and MO careers is shorter than that of MC. As additional data, only 6.5% of the students reported having some risk factor for the development of severe COVID-19. (Table 1)

**Table 1:** Demographic analysis of trained population

Total students	100%	616
Campus		
Monterrey	60.47%	372
Mexico City	27.85%	172
Guadalajara	11.68%	72
Year		
Fifth year	62.82%	389
Sixth year	37.17%	229
Degree		
Medical Surgeon (%)	89.3%	550
Nutrition and Integral wellbeing (%)	8%	49
Odontology (%)	1.8%	11
Risk factors for COVID-19		
No (%)	93.5%	576
Yes (%)	6.5%	40

**Figure 2:**
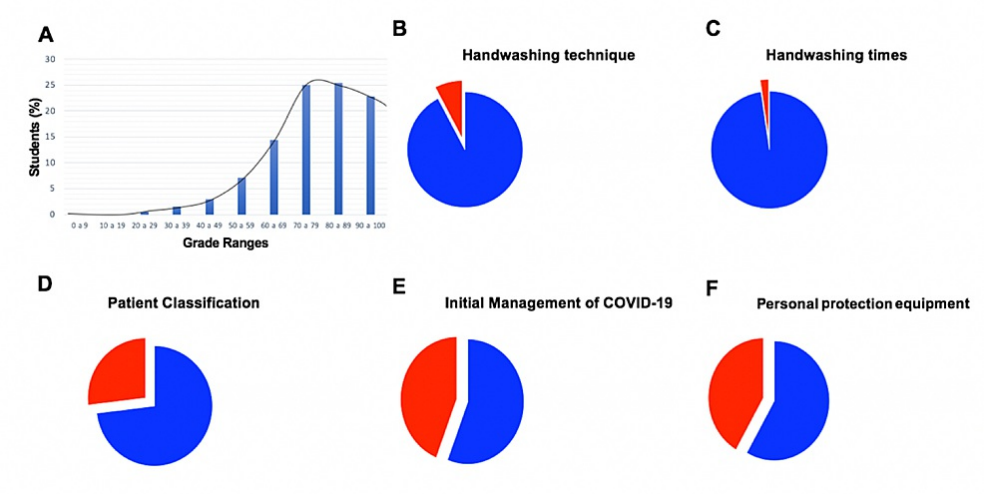
Results of the diagnostic evaluation of the clinical science induction training program. (A) Distribution of grades in the initial evaluation showing a mean of 77% of correct answers (SD ± 14.97), n = 570. (B-F) Exploration of in-hospital skills management in a health emergency situation. (B) Mastery of the handwashing technique (C) Mastery of the concept of the five moments of handwashing. (D) Identification and classification of patients with respiratory disease (E) Mastery of the approach and initial management of the patient with suspected COVID-19. (F) Proper handling of personal protective equipment. N = 570. Adequate domain is depicted in blue and inadequate domain in red.

A total of 570 from the total 616 students participated in the initial evaluation, with a mean of 77% correct answers (SD ± 14.97). (Figure 2A). The remaining 46 students were excluded from this analysis since they did not complete this first evaluation. The questions explored four main areas: hand-washing technique and times for hand-washing (The five moments for health hygiene proposed by WHO), classification of patients with respiratory disease, initial management of the patient with COVID-19, and management of PPE. It was observed that in the first area there was adequate theoretical domain of the subject, with 92% and 97% of correct answers for technique and times for hand-washing, respectively (Figures 2B-C). Regarding the classification of patients with respiratory disease, a lesser domain of the subject began to be observed, finding only 73% of correct answers (Figure 2D). Finally, in the last two areas, the domain was lower, with 41% and 44% incorrect answers, respectively (Figures 2E-F).

**Figure 3:**
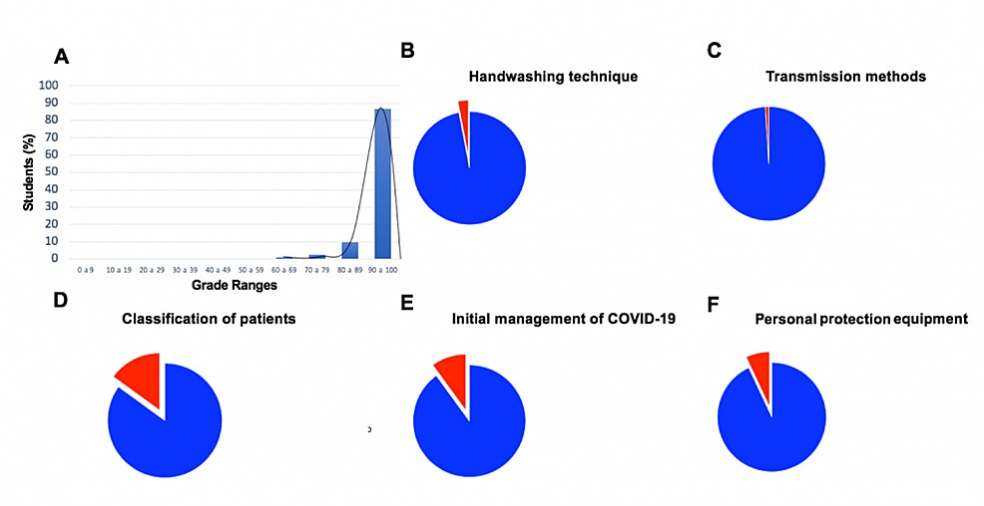
Results of the final evaluation of the clinical science induction training program. Distribution of grades in the initial evaluation showing a mean of 96% correct answers (SD ± 7.32), n = 616. (B-F) Exploration of in-hospital skills management in a health emergency situation (B) Mastery of the handwashing technique (C) Mastery of the concept of the transmission mechanisms of SARS-CoV-2. (D) Identification and classification of patients with respiratory disease (E) Mastery of the approach and initial management of the patient with suspected COVID-19. (F) Proper handling of personal protective equipment. N = 616. Adequate domain is depicted in blue and inadequate domain in red.

**Figure 4:**
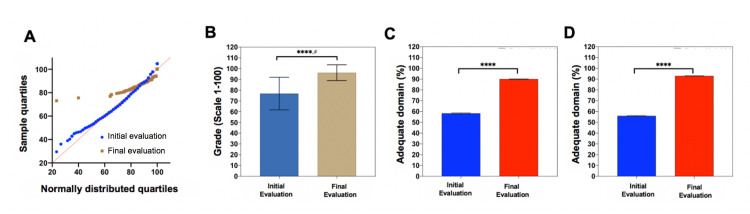
Global results of the impact of the training program for induction to clinical sciences. (A) Q-Q graph showing the differences between the distribution of the initial and final assessment scores compared to the Gaussian normal distribution. It was demonstrated that there was a non-normal distribution using the Kolmogorov-Smirnov test (KS distance = 0.0188) (B) Comparison of the means corresponding to the initial and final evaluations with Wilcoxon's W. (C) Comparison of the proportions with adequate mastery in the initial management of the patient with COVID-19 in the initial and final evaluation. (D) Comparison of the proportions of adequate mastery of the technique of use of personal protective equipment in the initial and final evaluation.  n1 = 570, n2 = 616. **** p≤ 0.001 # Cohen's D test with a statistic of 1.66 shows a high impact of the intervention.

Due to limitations in the design of the evaluations, it was not possible to perform descriptive statistics by career. 616 students participated in the final evaluation, with a mean of 96% correct responses (SD ± 7.32), showing a significant difference concerning the initial mean (p <0.001) and observing that the impact of the evaluation was high (d 1.6) (Figures 4A and 4B). Compared to the initial evaluation, in this assessment, all students completed the evaluation form in totality and none of the students were excluded from the analysis. The questions explored the same areas as in the initial assessment, but an additional one was added: COVID-19 transmission mechanisms, where a 99% dominance was observed (Figure 3C). In the rest of the areas, a significant improvement was found, finding a domain greater than 90% in all except in the classification of the patient with respiratory disease, where 15% had an inadequate domain, representing an area of ​​opportunity. (Figures 3B and 3D-F). To compare the results from initial and final evaluations, a two-sample proportion test was conducted, considering the differences in number of students at initial and final evaluation. A significant change was observed between the proportions of students who had adequate mastery in the final evaluation compared to the initial one in the areas of initial management of patients with COVID-19 (p <0.001) and management of the EPP (p <0.001), finding an increase greater than 31% and 37%, respectively. (Figures 4C-D) Therefore, it can be seen that although PPE management was the area with an unsatisfactory performance initially, it was also the one that saw the greatest improvement.

Results from the face-to-face practical examination regarding the correct use of PPE are summarized in Table 2. All tasks evaluated were registered in checklists by faculty (the results of the practical examination are independent of the overall results in the final evaluation). Overall, a high percentage of the students showed correct management and performance of the required skills and techniques for adequate PPE use within their first evaluation attempt. In this regard, the areas that possessed the highest levels of proficiency were correct opening, use and disposal of masks (99.67%), and appropriate hand hygiene after PPE removal (97.40%), while the lowest levels of proficiency were found on items regarding the correct order of PPE use (90.90%) and correct order and technique for glove removal.

**Table 2:** Assessed items and their overall results during the evaluation for the adequate use of PPE. Results are expressed in the number of students and their respective percentage that performed correctly the associated procedures during first attempt of simulation. Abbreviations: PPE: Personal protection equipment.

Assessment items	Students correctly doing (%)	Students incorrectly doing (%)
Correct handwashing technique	598(97.08%)	18(2.92%)
Correct order of PPE (gown, mask and gloves)	560(90.90%)	56(9.10%)
Correct mask opening and use	614(99.67%)	2(0.33%)
Correct tying of the gown	600(97.40%)	16(2.6%)
Correct order and technique of glove removal	556(90.25%)	60(9.75%)
Appropriate gown removal	570(92.53%)	46(7.47%)
Appropriate hand hygiene after PPE removal	600(97.40%)	16(2.6%)

A satisfaction survey was carried out that was, in general terms, favorable. The following areas were evaluated: planning, content, relevance for clinical fields, quality of technological resources, PPE workshop, speaker performance, and global evaluation. Each of these aspects were evaluated by the students using a Likert scale from 0 to 5, with 5 as the best performance. Six of the areas were rated positively by an average grade greater than 4 by those surveyed. A notable exception was in the quality of technological resources, which was the area with lower performance (3.7[±0.88]). The latter is partially explained by the speed with which the platform had to be designed as it was an emergency, but it represents an area of ​​opportunity for future interventions. Results from the satisfaction survey are summarized in Table 3. 

**Table 3:** Results from the satisfaction survey. Assessed items were evaluated by using a Likert scale (0 to 5), being 5 the highest grade possible and associated with the best performance. Results are presented as average and ± standard deviation.

Assessed items	Likert scale average (±SD)
Overall impression of the quality of the course planning.	4.5(±0.83)
Overall impression of the quality of the course content.	4.1(±0.53)
Impression about the relevance of the course for its application in the clinical fields.	4.6(±0.68)
Impression of the quality of technological resources.	3.7(±0.88)
Overall impression of the quality of the workshop on personal protective equipment.	4.7(±0.33)
General impression of the performance of the speakers.	4.7(±0.33)
Overall perception of the course.	4.4(±0.27)

**Figure 5:**
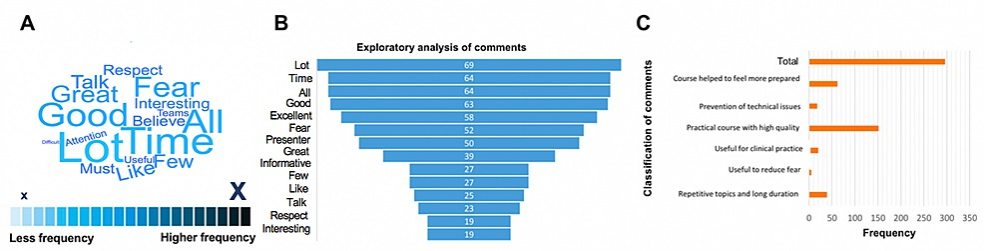
Qualitative analysis of the feedback comments from the trained group. (A) Word cloud scheme showing the frequency of words used in comments represented in color code and font size (see scale). (B) Frequency of the most relevant keywords in the exploratory analysis (only the 14-most frequent are represented). (C) Conceptual classification of feedback comments. N=297 (note: only a total of 297 students used this space to provide the comments since it was optional).

In the last question of the satisfaction survey, students were provided with an open space with a maximum of 300 words to explain their general comments, impressions, and suggestions for future educational interventions. A qualitative analysis of the feedback comments received by students was also carried out, where several positive qualifying adjectives were found among the most used words. However, a high frequency of words related to fear was also found, reflecting a potential area of ​​intervention through psychological support (Figures 5A-B). Finally, this analysis was also useful to detect other negative aspects that were not quantitatively observed; for example, the significant frequency of respondents whose impression was that some topics and presentations were repetitive and/or of long duration, possibly again representing the haste with which the course had to be designed (Figure 5C).

## Discussion

To the extent of our knowledge, the development of hybrid intervention models that would allow students in the health area to be trained to safely reintegrate into their clinical activities has been scarcely studied. Therefore, evaluating the impact of the training model described here is essential for the development of future interventions. The results of the initial evaluation demonstrate that the students had an acceptable mastery in various areas required for their daily clinical activities, such as the technique and five moments of hand washing. These results are in contrast to that reported in other studies in which only a small percentage of them have an adequate domain in this area [22]. On the other hand, the results observed in other aspects such as the management of PPE were alarming, being the area with the worst domain despite being essential for its safe clinical performance under the current context. These results showed that a face-to-face intervention was necessary to improve this particular competence.

The overall impact of this intervention turned out to be very satisfactory, as it demonstrated a substantial increase in the final averages concerning the initial ones, as well as in the domain of each one of the evaluated areas. It can also be observed that the areas where the worst performance was initially bad, such as the management of PPE and the initial management of the patient with COVID-19, were those that showed the greatest improvement, possibly due to the support of the face-to-face practical workshop, which addressed these aspects. Additionally, the results of the satisfaction survey, as well as the qualitative analysis of the students' comments, showed that the design and implementation of this model were acceptable. The perception of high applicability of the contents stands out, which contrasts with other interventions where one of the main drawbacks has been the complexity and lack of clinical applicability [23]. Additionally, this intervention had higher rates of student acceptance compared to previously published interventions, which used a virtual model [9, 24].

This program also showed challenges and areas of opportunity. It was shown by the satisfaction survey that a critical problem with this intervention was the poor quality of the technological resources employed. Similarly, other virtual medical education interventions have also referred to these limitations [9, 25]. Also, there was a significant perception that the sessions were of excessive duration and repetitive content, therefore, for the development of future interventions, they are required greater planning efforts and content delimitation. Possibly the development of a more specific course and with more practical activities have a greater impact on students. Although some studies have reported a high perception of fear of contagion associated with returning to clinical activities within medical students [24, 26], this intervention did not make an evaluation aimed at the psychological response of the students to the stress generated by their reintegration to clinical activities and represents an area of ​​opportunity for the development of future research.

## Conclusions

The COVID-19 pandemic has generated fundamental challenges in health education; however, it has allowed innovative tools and strategies to guarantee academic continuity. It is a fact that face-to-face clinical training cannot be substituted; therefore, the educational model for undergraduate students of health courses had to be modified during this adverse situation. Educative institutions must have a model to assure continuity of quality learning and reintegrate into clinical activities. In this sense, the hybrid model of intervention that was developed and evaluated in this study allowed students to be trained with theoretical knowledge and basic practical skills to let them face hospital environments with more security and confidence, reducing the possibility of adverse outcomes due to their integration. 

We conclude that this model is adaptable to any similar situation that modifies the normality of clinical education. Our model has high applicability; however, some areas of opportunity have been detected, so they should be considered when implementing it in other learning environments. Given the positive results even with many trained participants, the training model described here can help to develop future interventions and benefit countries where medical students have not restarted their activities within clinical environments due to the sanitary emergency. Based on a hybrid model, study plans could be redesigned to achieve the development of practical skills under the context of COVID-19 or future pandemics. With this, it would be possible to reduce the dissociation between theory and practice while obtaining better-trained individuals.
